# Diverse Rice Landraces of North-East India Enables the Identification of Novel Genetic Resources for *Magnaporthe* Resistance

**DOI:** 10.3389/fpls.2017.01500

**Published:** 2017-08-29

**Authors:** Bangale Umakanth, Balija Vishalakshi, P. Sathish Kumar, S. J. S. Rama Devi, Vijay Pal Bhadana, P. Senguttuvel, Sudhir Kumar, Susheel Kumar Sharma, Pawan Kumar Sharma, M. S. Prasad, Maganti S. Madhav

**Affiliations:** ^1^Biotechnology Division, ICAR-Indian Institute of Rice Research Hyderabad, India; ^2^Plant Breeding, ICAR-Indian Institute of Rice Research Hyderabad, India; ^3^Hybrid Rice Division, ICAR-Indian Institute of Rice Research Hyderabad, India; ^4^Plant Breeding Section, ICAR Research Complex for NEH Region, Manipur Centre Imphal, India; ^5^Plant Pathology Section, ICAR Research Complex for NEH Region, Manipur Centre Imphal, India; ^6^Plant Pathology Division, ICAR-Indian Institute of Rice Research Hyderabad, India

**Keywords:** rice landrace, blast resistance, genetic diversity, population structure, core set, association mapping

## Abstract

North-East (NE) India, the probable origin of rice has diverse genetic resources. Many rice landraces of NE India were not yet characterized for blast resistance. A set of 232 landraces of NE India, were screened for field resistance at two different hotspots of rice blast, viz., IIRR-UBN, Hyderabad and ICAR-NEH, Manipur in two consecutive seasons. The phenotypic evaluation as well as gene profiling for 12 major blast resistance genes (*Pitp*, *Pi33*, *Pi54*, *Pib*, *Pi20*, *Pi38*, *Pita2*, *Pi1*, *Piz*, *Pi9*, *Pizt*, and *Pi40*) with linked as well as gene-specific markers, identified 84 resistant landraces possessing different gene(s) either in singly or in combinations and also identified seven resistant landraces which do not have the tested genes, indicating the valuable genetic resources for blast resistance. To understand the molecular diversity existing in the population, distance and model based analysis were performed using 120 SSR markers. Results of both analyses are highly correlated by forming two distinct subgroups and the existence of high diversity (24.9% among the subgroups; 75.1% among individuals of each subgroup) was observed. To practically utilize the diversity in the breeding program, a robust core set having an efficiency index of 0.82 which consists of 33 landraces were identified through data of molecular, blast phenotyping, and important agro-morphological traits. The association of eight novel SSR markers for important agronomic traits which includes leaf and neck blast resistance was determined using genome-wide association analysis. The current study focuses on identifying novel resources having field resistance to blast as well as markers which can be explored in rice improvement programs. It also entails the development of a core set which can aid in representing the entire diversity for efficiently harnessing its properties to broaden the gene pool of rice.

## Introduction

Rice is a major staple crop in the world and demand for rice is increasing every year (Ray et al., 2013). Biotic stresses continue to be the constraint in rice production and becoming severe in the climate change regime. One of the most identifiable major biotic stresses is the blast disease caused by *Magnaporthe oryzae*. It infects rice leaves, nodes, color, and panicles at different stages of crop growth thus, decreasing the overall yield. In India, neck blast is becoming severe in many agro-ecological zones and causing more threats to the rice production (Laha, 2017). Although 100 major blast resistance genes (R-genes) have been identified, mapped and their tightly linked DNA markers are available (Miah et al., 2013), only one major gene (*Pb1*) has been reported for neck blast (Hayashi et al., 2010). In addition to this, rapid evolution and high variability of *Magnaporthe* with the rise in temperatures relates to its high pathogenicity. Hence identification of durable resistance sources and resistance genes is a continuous process in rice improvement programs.

Landraces are genetically dynamic and display equilibrium with both the environment and pathogens (Harlan, 1975). Many potential landraces of rice are being replaced by high yielding varieties to meet the food requirements. Despite being less productive they are known to have a high genetic variance for several biotic stresses (Hanamaratti et al., 2008), so they can be explored for rice improvement. North-East (NE) India, known to have highly diverse rice germplasm and the landraces belonging to this region are expected to have high genetic variability for many biotic stresses including the rice blast. Many rice landraces of NE India were not yet characterized for blast resistance.

Genetic diversity among the populations and their genetic relationships aids in conservation and parental selection in the improvement programs. Identification of populations with a high level of genetic variation will become a valuable resource for broadening the genetic base since it enables the identification of superior alleles for several traits including blast resistance. Association mapping is the best approach to identify the chromosomes segments harboring genes/QTLs (quantitative trait loci) controlling key traits and the genotype–phenotype associations in germplasm. For the successful application of association mapping, understanding of population structure is essential to avoid type I and type II errors between molecular markers and traits of interest (Zhang P. et al., 2011). Determination of population structure also helps in categorizing populations into subsections which are genetically related. The rapid advances in marker technology along with morphological traits can precisely estimate the genetic diversity among the populations (Kumbhar et al., 2015). For estimation of genetic diversity, population structure, linkage disequilibrium, and mapping various traits, molecular markers have been successfully used by many researchers (Ried et al., 2011; Nachimuthu et al., 2015; Anandan et al., 2016). SSRs aid in accurately estimating the genetic diversity among germplasm and are reported to be more efficient than single-nucleotide polymorphic markers (SNPs; Das et al., 2013; Singh et al., 2013; Nachimuthu et al., 2015). Development of a core set representing the total variation of the population with novel and superior traits is essential for successful utilization of the population. Core and mini core sets of rice were earlier made using morphological traits and molecular markers (Zhang H. et al., 2011; Liu et al., 2015). However, no core set is available exclusively for the NE landraces. Evaluation of genetic diversity among NE Indian germplasm was reported earlier (Das et al., 2013; Roy et al., 2014, 2015) but very few studies exist on the understanding the diversity in relation to the blast resistance (Ghaley et al., 2012; Mahender et al., 2012).

In the current study, we have systematically performed phenotyping for blast resistance in a span of two years. Phenotyping along with gene profiling for major blast genes identified novel genetic resources which might contain the novel resistance genes. For clear understanding, the genetic diversity of NE landraces were analyzed with distance as well as model based diversity approaches. To apply the existing diversity in breeding programs, a core set was derived. Using 232 landraces, the marker–trait association was identified for blast resistance as well as yield-related traits. The information generated in the present study has a lot of value in the current rice improvement programs.

## Materials and Methods

**FIGURE 1:**
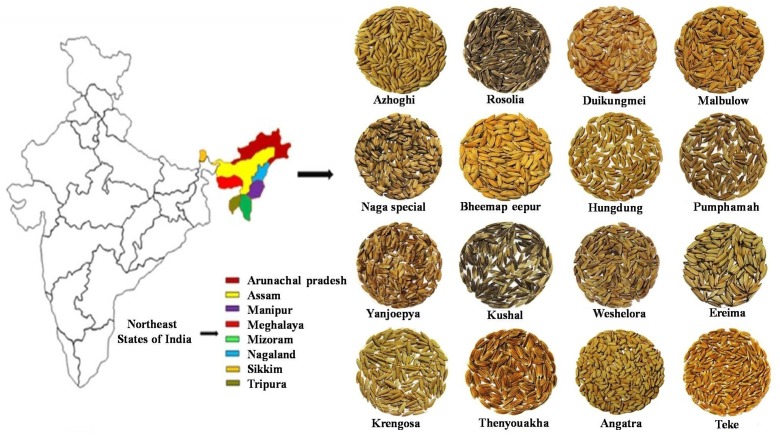
Geographical distribution of diverse rice landraces (232) across North Eastern states of India.

A total of 232 landraces (Supplementary Table S1) were collected from the NE region of India (**Figure 1**) for the current study. Along with the landraces, three varieties of Basmati, five varieties of Indica, seven varieties of Temperate Japonica, and five varieties of Tropical Japonica were also selected for the comparison of landrace alleles. The line harboring particular blast resistance gene was considered as a positive control, and the susceptible cultivars namely BPT, Swarna, and CO39 which do not have any blast resistant gene were considered as negative controls for gene profiling.

### Evaluation of Blast Disease at Two Hotspot Locations

Approximately 40–50 seeds of each landrace were sown in uniform blast nursery (UBN) for phenotyping of leaf blast disease at ICAR-IIRR, Hyderabad, India. The entire nursery block, as well as each entry, was surrounded by HR12 (susceptible cultivar) on four sides for spreading the disease evenly. A mixture of *M. oryzae* isolates collected from different blast hotspot regions of Andhra Pradesh and Telangana (Surapu et al., 2017) were used to screen landraces for the leaf blast disease. The plants were spread with inoculum at the two-leaf stage and the score was taken once the susceptible check gets totally infected with blast disease. The score was taken based on 0–9 scale (Standard evaluation system, SES, IRRI, 2002; Prasad et al., 2011). Scores 0–3 were considered resistant (R), 4–5 as moderately resistant (MR), and 6–9 as susceptible (S). Field screening of blast disease was done at ICAR Research Complex for NEH Region, Manipur under natural disease conditions and the disease pressure was increased by inoculating with the mixture of *Magnaporthe* isolates that were collected from different blast hotspots of NE India. For augmenting the disease pressure for Neck blast, sowings were delayed than usual season and also each plant was injected with a spore suspension of a mixture of isolates collected from NE India using a syringe (Aglawe et al., 2017). Disease score for each plant was recorded for at least three times and the highest score out of these was considered. Neck blast was evaluated based on the percentage of infections on the neck of the panicle at physiological maturity of the plant (Madhav et al., 2013). The highest disease score observed in both the seasons was considered. The screening was done in the same way for two seasons (Kharif 2012 and 2013) in two replications.

#### Agro-morphological Evaluation of Landraces

The landraces were grown in a block prepared by uniform distribution of soil enriched with nutrients required for the plant growth. A spacing of 20 cm between each row and 15 cm between plants was adopted at the time of planting. Twenty plants of each landrace were sown in rows and the data was recorded for five selected plants. Data was taken on various parameters like plant height, presence and absence of awns, seed color, tiller number, and spikelet number. At the time of harvesting, biomass and yield per plant were also recorded.

#### DNA Isolation

DNA was isolated from the leaf tissue by following Murray’s (Murray and Thompson, 1980) protocol. The quantity of DNA was estimated in nanodrop (Thermo Fisher Scientific, United States) as well as in agarose gel.

#### Gene Profiling

PCR analysis was done for landraces together with the positive controls and susceptible checks to identify the presence of 12 major known blast resistance genes namely *Pitp*, *Pi33*, *Pi54*, *Pib*, *Pi20*, *Pi38*, *Pita2*, *Pi1*, *Piz*, *Pi9*, *Pizt*, and *Pi40* using molecular markers as described in Devi et al. (2015). Gene-based (SNP and STS) markers were used for six genes, viz., *Pi54*, *Pi40*, *Pita2*, *Pi9*, *Piz*, and *Pizt* and the remaining genes were profiled with tightly linked SSR markers. The markers used for the gene profiling and positive controls for respective genes are listed in Supplementary Table S2. Genetic diversity analysis was carried out with 120 SSR markers which are uniformly spread across the 12 chromosomes (Supplementary Table S3).

#### Diversity Analysis

For diversity analysis, all the alleles derived from each SSR marker were scored as “1” for the presence of amplicons and “0” for its absence in the respective allele position. The size of the amplicons was also determined based on their migration relative to standard molecular weight marker (50 bp DNA ladder from Fermentas). Allele number, allele frequency, heterozygosity, and polymorphic information content (PIC) values for all the markers were calculated using Power marker V3.25 software (Liu and Muse, 2005).

### Clustering of Genotypes

For clustering of genotypes, a pairwise distance matrix was computed using DARwin V6 software. An unweighted neighbor-joining tree was constructed using dissimilarity index method. The genetic distance was calculated by Jaccard’s coefficient with bootstrap analysis of 1000 iterations using the SSR marker data (Perrier and Jacquemoud-Collet, 2006).

### Population Structure Determination

The structure of the population was determined by a Bayesian-based approach using Structure V2.3.4 software (Pritchard et al., 2000). The number of subpopulations was classified based on the true *K* value. For accurate determination of subpopulations, admixture model was followed. In this model, the burnin period length was set to as high as 150,000 with Markov Chain Monte Carlo (MCMC) 150,000 replications. Each *K* value was run for 10 times with *K* value varying from 1 to 25. To obtain clear peak at the Δ*K* (*ad hoc* quantity) to *K* value, the output generated from structure software was loaded in structure harvester (http://taylor0.biology.ucla.edu/structureHarvester/).

### Molecular Variance

The presence of molecular variance among the population and individuals estimated by structure was analyzed through analysis of molecular variance (AMOVA). The estimation of AMOVA based on Nei’s distance matrix (Nei, 1973) along with the pairwise *F*_ST_ and the principal component analysis (PCoA) to characterize the subgroups of the germplasm set was computed using GenAlEx 6.5 software (Peakall and Smouse, 2012).

### Establishment of Core Set

A mini-core collection was developed using Powercore V1.0 software (Kim et al., 2007). For both core and mini-core collections, Nei genetic diversity index and Shannon–Weaver diversity index was calculated. The significant difference percentage between core set and the entire collection was analyzed for evaluating mean difference percentage (MD%) and variance difference percentage (VD%) of traits. To evaluate the properties of a core set against the entire collection, the coincidence rate percentage (CR%) and variable range percentage (VR%) were evaluated.

### Association Analysis

The association tests between the marker and the agronomic traits in the population were run based on two models, general linear model (GLM) and mixed linear model (MLM) using TASSEL V3.0 (Bradbury et al., 2007). GLM was run by considering the population structure of accessions as described by Kang et al. (2008). The MLM was performed using the Kinship file generated from genotypic data and Q matrix generated by running population structure at optimized *K* value in order to minimize the false positive associations (Yu et al., 2006; Gupta et al., 2014). The significant marker–trait association was determined by *P* ≤ 0.05 and the magnitude of the QTL effects by marker *R*^2^.

## Results

### Leaf and Neck Blast Phenotyping

The stringent blast screening of 232 landraces at two locations in the year 2012 revealed 166 landraces showing resistance to leaf blast and 148 to neck blast. Whereas in 2013, 172 landraces were resistant to leaf blast and 154 to neck blast. On average, 153 landraces showed resistance to leaf blast and 135 landraces resistance to neck blast and interestingly, 91 showed resistance to both leaf and neck blast. The mean scores of resistant landraces ranged between 0 and 3 while the susceptible check HR12 gave the score of 9.

### Gene Profiling

**FIGURE 2:**
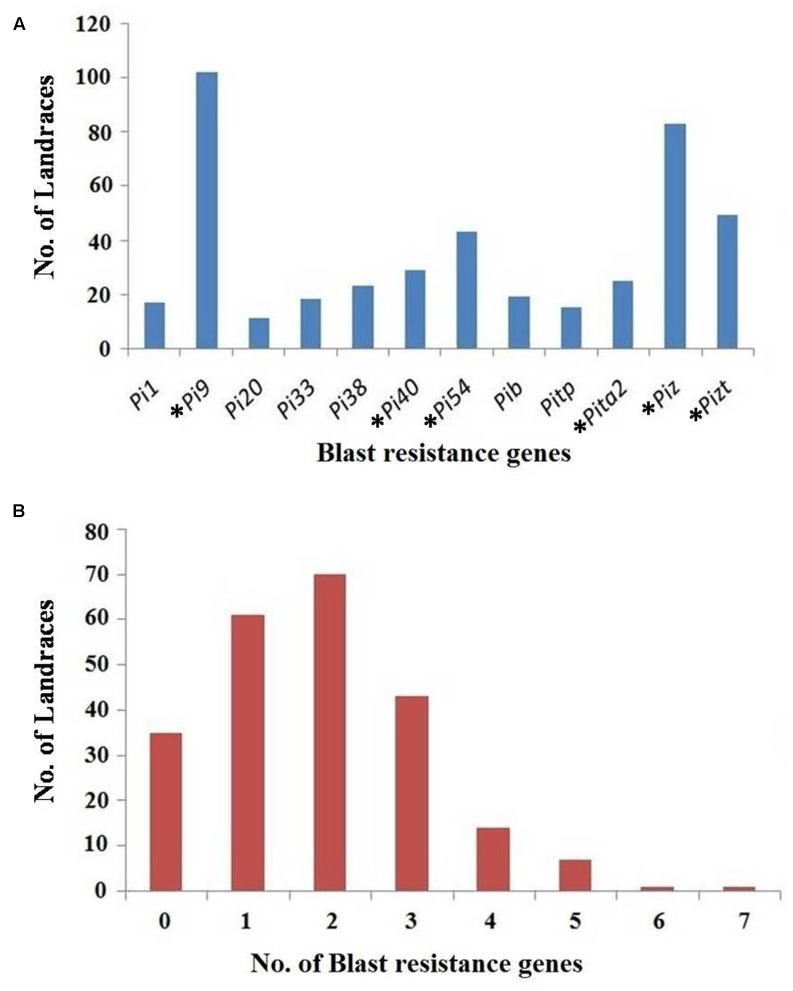
**(A)** Distribution of 12 major blast resistance genes in a set of 232 rice landraces collected from North-East region of India (^∗^gene having a gene-based marker). **(B)** Landrace harboring different number of blast resistance gene.

**FIGURE 3:**
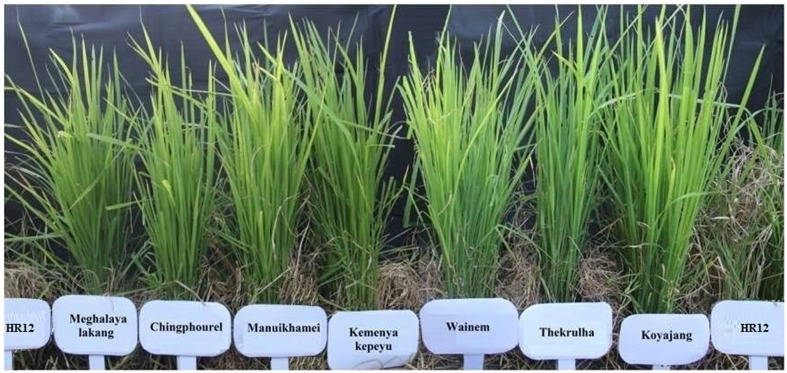
Phenotypic reaction of unique seven blast resistant landraces at IIRR’s uniform blast nursery with HR12 as a control for susceptibility.

The gene profiling of landraces revealed that *Pi9* was found in the maximum number of landraces, i.e., 102 (43.9%), while the *Pi20* gene is present in the least, i.e., 11 landraces (4.74%). One unique landrace Apaghi june found to be a rich source for blast genes as it has seven genes, viz., *Pi9*, *Piz*, *Pi33*, *Pib*, *Pi38*, *Pi40*, and *Pi54.* Another landrace, i.e., Chalhtssia has six genes, viz., *Pi1*, *Pi9*, *Pi54*, *Pib*, *Pitp*, and *Piz.* Seven landraces have the combinations of five genes whereas 14 landraces have four gene combinations. Similarly, the landraces containing three gene combinations, two gene combinations and single genes were also identified; these details are given in Supplementary Table S1 (**Figure 2**). Among the 91 blast resistant landraces, seven landraces, viz., Meghalaya lakang, Chingphourel, Manuikhamei, Kemenya kepeyu, Wainem, Thekrulha, and Koyajang do not have any of the tested major blast resistant genes (**Figure 3**).

### Genetic Diversity

Diversity analysis with the allelic data generated from 120 SSR markers detected a total of 360 alleles, as the alleles per loci ranged from 2 to 8 with an average of 3 alleles per locus. Among the total alleles, 15% rare alleles (having allele frequency <5%) were found. The SSR marker RM22837 (Chr-8) recorded the highest number of alleles (8), which indicates its high polymorphic nature, while many markers showed two alleles. The major allele frequency ranged from 0.31 to 0.98 with an average of 0.59 per locus, and the average observed heterozygosity was 0.09. Where as expected heterozygosity ranged from 0.05 to 0.80 with an average of 0.51 per locus. The relative informativeness of each marker has been identified by PIC value, highest PIC value (0.77) was found for RM22837 while least (0.05) for RM21052 with an average PIC value of 0.44 for markers (Supplementary Table S3).

**FIGURE 4:**
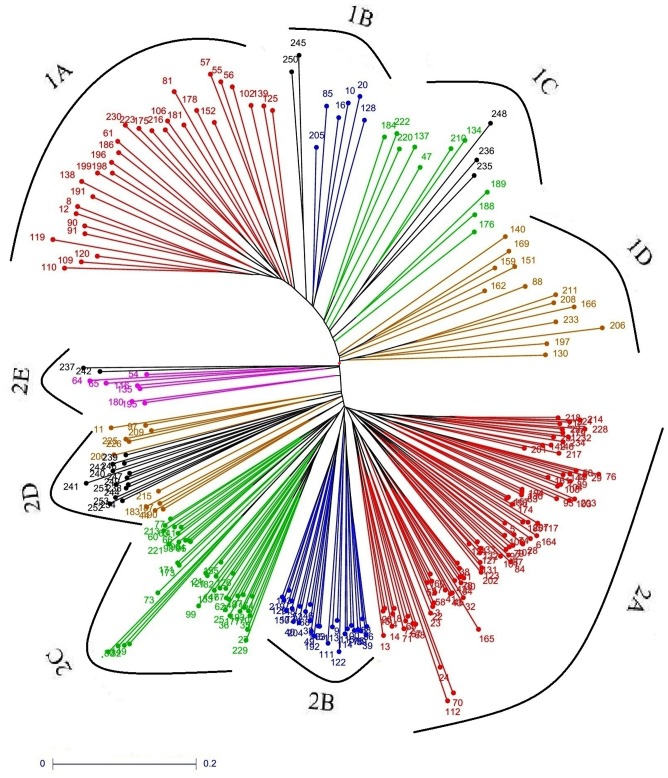
Unweighted neighbor-joining tree of landraces grouped into two major clusters representing the genetic relationship among the landraces.

The neighbor-joining tree showed the presence of two distinct major clusters, of which 59 landraces grouped in cluster-1 and 173 landraces in cluster-2. The cluster-1 is again divided into four subclusters—1A, 1B, 1C, and 1D, whereas cluster-2 divided into five subclusters, i.e., 2A, 2B, 2C, 2D, and 2E. The landraces present in the subcluster 1B shares alleles with Japonica and Tropical Japonica varieties while landraces present in 1C shares with Japonica and Basmati accessions. The landraces present in the subcluster 2D shares with Indica, Japonica, and Tropical Japonica varieties while landraces of 2E shares with Japonica and Basmati accessions. The sub-clusters 1A, 1D, 2A, 2B, and 2C formed exclusively with landraces (**Figure 4**).

**FIGURE 5:**
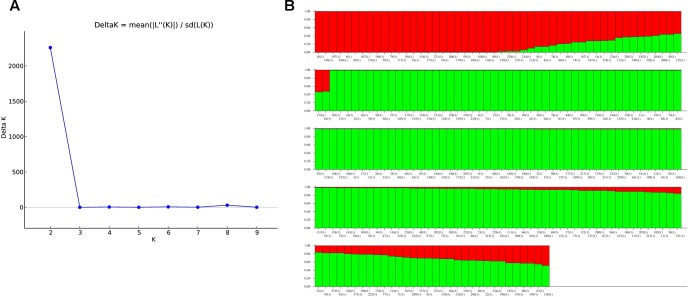
Representation of population structure dividing the landraces in two subgroups based on *K* value **(A,B)**. Landraces with >80% probability are assigned to corresponding subgroups and exceptions are shown as an admixture. **(A)** Population structure inferring the landraces divided into two subgroups based on *K* value. **(B)** Population structure inferring the landraces divided into two subgroups based on *K* value. Landraces with probability >80% were assigned to corresponding subgroups and remaining as an admixture.

The population structure analysis of landraces revealed the log likelihood value (Δ*K*) maximized to the highest value of 2260.4 at *K* = 2 (**Figure 5A**), showing a clear peak indicating the classification of entire population into two distinct subgroups SG1 (subgroup1) and SG2 (subgroup2) (Supplementary Table S1). The landraces sharing the membership fractions with a probability of greater than 80% were allotted to corresponding subgroups (**Figure 5B**). Among the 232 landraces, 33(14.2%) landraces were categorized under SG1 whereas the majority of the landraces that is 158 (68.1%) were grouped in SG2, and the remaining 41(17.7%) landraces were grouped as admixture. Subgroup2 which has a high number of landraces (158) was further subjected to structure analysis. This analysis revealed the classification into four sub-subgroups (SSG) (Supplementary Figure S1) as the Δ*K* value showed a clear peak at *K* = 4. The SSG1 consists of 24 landraces, 13 in SSG2, 10 in SSG3, 12 SSG4, and 99 landraces in admixture.

The distribution of molecular variance exposed significant genetic differentiation between and within populations among the landraces. Molecular variation of 75.1% was observed among the individuals within the subgroup (population) and 24.9% variation was observed among the subgroups. The pairwise *F*_ST_ value 0.278 indicated the significant difference between two subgroups (**Table 1**).

**Table 1:** AMOVA between structured landraces and pairwise comparison using *F*_ST_ values (GenAlEX).

	AMOVA			
	
Source	Degrees of	Sum of	Variance	Percentage of
	freedom	squares	components	variation
Among the populations	2	854.725	3.921	24.90%
Within populations	229	6228.687	12.253	75.10%
Total	231	7083.412	16.174	100%

**Pairwise population *F*_ST_ values**				

	**SG2**	**AD**		

SG1	0.27	0.096		
SG2	–	0.093		


The PCoA was performed for characterizing the subgroups of landraces. The first two-dimensional scatter plots described the PCoA axes accounting for 12.48 and 4.04% of the genetic variation among subpopulations formed from 252 accessions (Supplementary Figure S2).

### Powercore Analysis

**FIGURE 6:**
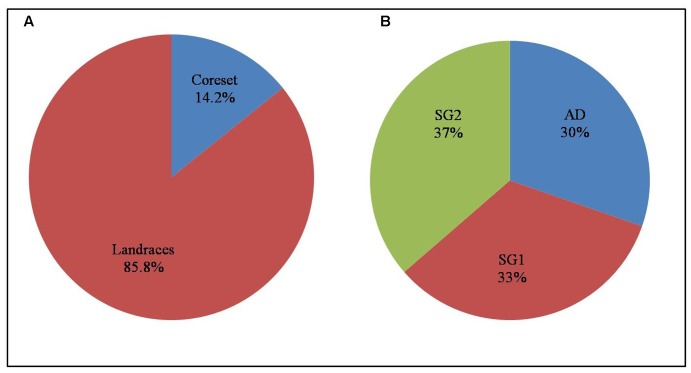
Depiction of **(A)** core set land races and **(B)** distribution of its subgroups.

To make the core set representing the entire diversity of landraces, 11 morphological characters and genotypic data of 120 SSR markers was used for the power core analysis using non-heuristic approach. This analysis identified 33 landraces (14.2%) that represent the core set (**Figure 6** and Supplementary Table S4A). The core set has 4.42% MD%, and 100% CR%. The core collection with VD% 29.17% and VR% (variable rate of coefficient of variation) 118.18% was observed. The efficiency index of the core was 0.82 and the PIC was 0.99 estimated by phenotypic characters and SSR markers (Supplementary Table S4B). Among phenotypic traits, biomass contributed to the highest Nei’s diversity index of 0.74 while the presence of awn contributed the lowest (0.21). The SSR markers showed an average Nei’s diversity of 0.6 in the core set.

### Association Analysis

To identify the marker associated with the blast resistance as well as with other traits, we used 232 landraces for association study. Association analysis of landraces based on GLM and MLM using Tassel software revealed 20 marker–trait associations with a significant *P*-value of less than 0.05. One marker (RM480) present on chromosome-5 showed association with leaf blast with a significant *r*^2^ value of 0.21 and at this location, blast gene *Pi26* was reported. Similarly, RM5401 located on chromosome 5 having an *r*^2^ value of 0.21 is associated with the neck blast resistance where no single QTL for blast resistance was reported. Two markers (RM21936 and RM3787) located on chromosome 7 and chromosome 9, respectively were associated with both leaf and neck blast resistance. About 15 markers were associated with yield-related traits namely number of tillers, a number of spikelets, plant height, yield per plant, and color. The marker–trait associations and their significance were listed in **Table 2**. The relative kinship estimates based on the 120 SSR data resulted in 83.3% of the pairwise kinship estimates ranging from 0.25 to 1.0 and the remaining estimates ranged from 1 to 2, with a low number of pairs filling in the higher estimate categories (Supplementary Figure S3). The kinship analysis revealed that the many of the accessions had a weak relationship with the other accessions in the landrace population. The linkage disequilibrium analysis among the locus pairs of 120 SSR markers revealed the *D*′ values ranging from 0 to 0.52 with an average of 0.33. About 26% of the marker pairs were having a significant LD (*P* < 0.01).

**Table 2:** Association analysis between agronomic traits and SSR markers.

S. No.	Trait	Marker	Chromosome	Marker *P*	Marker *R*^2^
1	Leaf blast	RM480	5	0.0317	0.21
2	Neck blast	RM21936^∗^	7	0.00758	0.30
3	Neck blast	RM5401^∗^	5	0.03602	0.21
4	Leaf and neck blast	RM21936^∗^	7	0.00704	0.32
5	Leaf and neck blast	RM3787^∗^	9	0.02481	0.22
6	Number of tillers	RM6105	11	0.00199	0.15
7	Number of tillers	RM1358	2	0.0165	0.20
8	No of panicles	RM6105^∗^	11	0.00145	0.17
9	No of panicles	RM1358^∗^	2	0.00707	0.23
10	No of panicles	RM523	3	0.03796	0.19
11	Plant height	RM6051	9	0.01248	0.28
12	Plant height	RM286	11	0.01459	0.31
13	Plant height	RM432	7	0.03184	0.17
14	Grain yield	RM5499	7	0.00005	0.33
15	Grain yield	RM28404^∗^	12	0.01289	0.20
16	Grain yield	RM190	6	0.02427	0.15
17	Grain yield	RM3827	6	0.02729	0.22
18	Color	RM2136	11	0.00026	0.18
19	Color	RM6404^∗^	10	0.02377	0.15
20	Color	RM1227	12	0.02964	0.13


*^∗^Novel associations for respective traits.*

## Discussion

Successful rice breeding programs require the presence of genetic diversity in the existing germplasm. The current study focuses on ascertaining this phenomenon in NE Indian landraces. We used a completely unique and unexplored germplasm for identifying the best donors and markers associated with blast resistance. We demarcate the diversity into a manageable core set.

Systematic screening for blast disease at two different locations for two consecutive seasons led to the identification of 91 landraces resistant to both leaf and neck blast. Blast screening at different locations increases the consistency of phenotyping as different virulent blast isolates have diverse pathogenic behavior (Madan et al., 2012). Previously a similar strategy was used for identification of best introgression lines for blast resistance (Devi et al., 2015). A mixture of blast isolates from geographically distinct locations (Hyderabad and Manipur) led to the identification of stable QTLs in one of the NE landrace (Aglawe et al., 2017). Neck blast is becoming more problematic than the leaf blast at several locations in India (Aglawe et al., 2017; Laha, 2017) so, it has become imperative in breeding programs to choose the accession having resistance for two phases of blast resistance (Abhijeet et al., 2013). So far only one neck blast resistance gene (*Pb1*) was identified (Hayashi et al., 2010) and many of leaf blast resistant accessions may not hold the resistance for the neck blast, hence screening for neck blast was given a priority. Moreover, the NE hilly areas have compatible climatic conditions for a natural infestation of neck blast.

In addition to the phenotyping, gene profiling using SNP, SSR, STS, and indel markers was also performed so as to identify the presence of valuable genes to the blast resistance (Cho et al., 2008) (Supplementary Table S2). These results indicated that *Pi9* was major gene followed by *Piz* and *Pizt* existing in landraces. The *Pi9* is predicted to be persistent in landraces from a long time as it was originated from the wild species (*Oryza minuta*) of rice. Anupam et al. (2017) identified the frequency of seven blast genes ranging from 0 to 80% in 74 local landraces collected from Tripura by profiling with gene-based STS and SNP markers. Prevalence of *Piz* among 32 NE Indian landraces by gene profiling with STS and SNP markers was reported (Imam et al., 2014). Shikari et al. (2013) also evaluated the 54 landraces collected from different parts of India for the presence of *Pita* and *Pita2* genes with two gene-based and three linked markers and found 11 landraces containing *Pita* and nine landraces containing *Pita2* genes. Gene-specific markers of eight blast resistance genes (*Pib*, *Piz*, *Pizt*, *Pi9*, *Pi40*, *Pi5*, *Pia*, and *Pita*) were also explored by Mahender et al. (2012) for characterizing the various accessions of Manipur (another state of NE India) and found landraces containing two to seven blast resistance genes, which again indicates the richness of blast diversity in landraces of NE India. In the current study, the landraces used were entirely different from the previous work (Mahender et al., 2012; Imam et al., 2014; Roy et al., 2015), we also checked their presence in the national gene bank (National Bureau of Plant Genetic Resources, NBPGR, New Delhi) and found 189 landraces that does not exist in it. Around 85% of landraces (197) harbors blast genes ranging from 1 to 7, indicating the rich diversity for blast resistance and these landraces serves as good genetic resources for blast resistant genes. Interestingly, seven resistant landraces did not show the presence of any of the tested genes, may contain the novel genes (Supplementary Table S1 and **Figures 2**, **3**). Identification of new genes having durability and its transfer into a cultivable variety may increase its resistance toward the disease since most of the identified genes are losing their effectiveness to blast disease (Das et al., 2012).

Genetic diversity analysis was done for determining the diverse nature of the landraces. To precisely differentiate closely related individuals, 120 SSR markers which are well distributed in the genome were used in the current study. The extent of polymorphism was detected by calculating PIC values and for evaluation of genetic diversity across the chromosomes; an allelic number was also calculated. Average PIC value of markers was 0.44 with an average of three alleles per locus (Supplementary Table S3). An average PIC value of 0.47 was reported in 74 Tripura rice landraces by using 30 SSR markers (Anupam et al., 2017). Roy et al. (2016) found a PIC value of 0.62 with the NE Himalayan landraces. Yang et al. (1994) reported average PIC value of 0.58 in 238 landraces collected from India, China, Japan, and IRRI. Giarrocco et al. (2007) also found average PIC value of 0.69 in 68 accessions of Argentina. A significant amount of rare alleles were found (15%) which might contribute to the overall genetic diversity of the population. Nachimuthu et al. (2015) reported the presence of 5% rare alleles in 192 rice germplasm upon screening with 61 SSR loci. Anupam et al. (2017) reported the existence of 11 rare alleles in 74 germplasm lines upon analysis with 30 SSR loci.

In the present study, a gene diversity of 0.51 was observed among the total population (232 landraces), which is comparable to most of the diversity panel consisting of global accessions (gene diversity of 0.5–0.7) (Ni et al., 2002; Garris et al., 2005; Ali et al., 2011; Roy et al., 2015, 2016). Gene diversity of NE landraces is higher than overall gene diversity of rice core collection (0.544) from China, Philippines (Zhang H. et al., 2011) and US accession panel (average gene diversity of 0.43; Agrama and Eizenga, 2008). These results clearly indicating that the NE landraces of India are showing the high genetic diversity in rice that exists in India (Supplementary Table S3).

The distance based clustering analysis grouped the landraces into two major clusters (**Figure 4**). Choudhury et al. (2013) used the distance based clustering method to group the 100 cultivable rice varieties released in different states into their respective decade periods. Similarly, rice genotypes collected from Tamil Nadu were classified into *indica* and *japonica* types based on the distance based clustering (Vanniarajan et al., 2012). In addition to the distance based clustering of the population, the genetic architecture of diverse material was also done by the model based structure analysis, as it offers better architecture of population with molecular markers (Powell et al., 1996; Horst and Wenzel, 2007; Varshney et al., 2007). Several researchers have analyzed the population structure by implementing model-based approach using Structure for numerous rice accessions (Garris et al., 2005; Agrama et al., 2007; Zhang et al., 2007, Zhang P. et al., 2011; Agrama and Eizenga, 2008; Jin et al., 2010; Ali et al., 2011; Chakhonkaen et al., 2012; Courtois et al., 2012). The present study classifies the landraces into two subgroups SG1 and SG2 using the threshold value >80%. Similar results were observed in grouping 3024 Chinese rice landraces (Zhang et al., 2009), 64 landraces of NE Himalayan region of India (Roy et al., 2016), and 192 rice accessions of varied origin (Nachimuthu et al., 2015). But, several studies were reported on the grouping of rice accessions which varied from two to eight groups (Garris et al., 2005; Agrama et al., 2007; Zhang et al., 2007, 2009; Zhang P. et al., 2011; Ali et al., 2011; Chakhonkaen et al., 2012). Roy et al. (2015) divided 107 NE landraces into three different subgroups by structure analysis using 40 SSRs. Das et al. (2013) used 23 SSR markers and distinguished 91 rice lines (which include 83 NE landraces and eight checks) into four subpopulations. Thirty-seven chakhao landraces of NE were also divided into six subgroups based on genetic structure analysis (Roy et al., 2014). The current study grouped 232 accessions based on the maximum membership probability of >80%, into 33 accessions under SG1, 158 accessions under SG2 and 41 accessions as an admixture (**Figure 5**). Further population structure analysis in SG2 classified the landraces into four subgroups indicating that independent structure analysis defines the precise genetic structure of the accessions. The results of the unweighted neighbor-joining tree constructed on the basis of UPGMA and PCoA harmonized with the structure analysis of the landraces. The PCoA cited the resistant and susceptible genotypes into four different quadrants at different positions.

AMOVA is useful for studying molecular variance within the species. It is used to study the patterns and degree of relatedness revealed by multidimensional scaling and the clustering dendrogram. It also summarizes the population structure, while remaining flexible enough to accommodate different types of assumptions about the evolution of the genetic system. AMOVA of the population showed greater variability among the individuals within the populations. The variation observed among the individuals within populations was higher (75.1%) than the variation present between the populations (24.9%). The *F*_ST_ value of 0.27 indicated that the higher divergence was observed between subgroups. Nachimuthu et al. (2015) upon analyzing 192 rice accessions (landraces and varieties collected from different parts of the world) and grouped them in two subgroups and reported 14% variations among the groups and 86% within the group. Similarly, Zhang et al. (2009) also grouped the 3024 rice accessions, into two subgroups and found the variation among the groups which was little higher (36.65%) and variation among individuals within populations was 40.80%.

The development of core set is very much essential for better management of germplasm lines in crop improvement strategies. It is helpful to precisely characterize, explore and conserve resources, monitor the genetic drift during preservation and identify gaps in genetic diversity (Frankel and Brown, 1984). Choudhury et al. (2014) developed a core set of 701 accessions (10%) among 6984 germplasm lines collected from NE India using molecular markers and agronomical traits. Zhang H. et al. (2011) established a core set of 189 accessions from 4310 Chinese accessions which include landraces. In the present study, a core set of 14% (33 landraces) was developed which represents the existing entire genetic diversity of the total landrace collection (Supplementary Table S4A and **Figure 6**). The core set developed indicates the homogeneous distribution ranges of the phenotypic traits as MD% is less than 20% and CR% is larger than 80% (Kim et al., 2007). The VD% and VR% present within the range represents the diversity as the original collection (Hu et al., 2000). This shows that the core collection represents the entire collection. At present, there is no universally accepted method for the development of core set as various factors such as sampling percentage, data type, number of traits observed, genetic diversity of germplasm, grouping method, and sampling method will play in demarcating the core group (Upadhyaya et al., 2006; Diez et al., 2012; Rao et al., 2012).

Association mapping is a novel approach which helps in identifying marker–trait relation based on linkage disequilibrium (Zondervan and Cardon, 2004). Based on GLM, we identified 20 marker associations for blast disease and other agronomic traits. Of which, eight markers loci are novel since, in their location, QTLs for those traits were not reported. These newly identified trait-associated SSR markers can be used for the discovery of novel genes/QTLs. While with MLM analysis, only 17 marker–trait associations were found and all these associations were reported in the GLM. Associations detected in GLM were not found in MLM, since in later analysis effect of kinship may play a role. The markers RM5499 and RM190 which are previously reported for grain yield also showed association in the present study and in the same way two markers (RM286 and RM6051) for plant height. Few markers showed association with more than one trait which is of great interest in breeding programs. Two markers RM21936 and RM3787 were newly identified to be associated significantly (*P* < 0.05) with leaf and neck blast disease (**Table 2**). These markers may be helpful for mapping genes for neck blast with durable resistance which does not exist in the currently available germplasm. Exploring novel gene pool for neck blast resistance and identification of novel genes which can offer resistance to leaf and neck blast resistance has become quite essential. Anandan et al. (2016) reported 16 marker associations in 629 rice genotypes using 39 SSR markers for identification of genes associated with early seedling vigor using GLM. In an association analysis of yield and grain quality traits in Embrapa Rice Core Collection (ERiCC) of 242 accessions (Inbred lines, cultivars, upland, and lowland accessions) from Brazil with 86 SSR markers and field data, eight markers were identified which were associated with four different traits (Borba et al., 2010). Twenty marker associations were identified for 29-grain metabolites in rice using 218 SSR markers in a set of 48 rice germplasm lines from the Chinese core collection (Lou et al., 2011). Twenty-six SSR markers associated with blast resistance (*P* < 0.01) in a set of 276 indica landraces from China and few from different parts of the world were reported (Wu et al., 2016). The identified markers associated with specific traits can be validated for their effectiveness in various genetic backgrounds and those can be helpful to the breeders in the pyramiding of QTLs from different sources through marker-assisted selection. Hence association mapping became an unleashing tool for identification of markers linked to different traits.

## Conclusion

Host and pathogen arms race is a continuous phenomenon in evolution. The premise of collecting, analyzing and characterizing unexplored NE landraces is for identification of donors containing multiple blast resistance genes which can be readily used in the breeding programs. The unique seven landraces (Meghalaya lakang, Chingphourel, Manuikhamei, Kemenya kepeyu, Wainem, Thekrulha, and Koyajang) having resistance to leaf and neck blast can be explored for the discovery of novel genes for broadening the gene pool to combat the pathogen competition. Distance and model based diversity analysis are highly correlated and genetic architecture of diverse material was effectively determined. The rich diversity existing in the landraces were divided into manageable subgroups and identified the molecular variance present among the populations and individuals which can be harnessed in the selection of distant individuals. To utilize the diverse landrace germplasm, an efficient core set which represents the entire diversity was made. Further, association analysis using a core set identified novel eight markers associated with the blast and other agronomically important traits, these markers are the candidates for the validation in the larger populations. The information emanating this work will help in conservation and utilization of valuable rice germplasm of NE India.

## Author Contributions

MSM designed all experiments, developed the structure and arguments for the paper, made critical revisions, and approved the final version. BU and BV carried out the major work and prepared manuscript and contributed to the refinement of the manuscript. PSK helped in analyzing the data and SRD helped in data collection of agro-morphological traits. PS reviewed and approved the final manuscript. VPB, SK, SKS, and PKS helped in the collection and phenotypic screening of rice landraces for neck blast resistance at the North-East region. MSP helped in the screening of leaf blast resistance at IIRR. All authors have read and approved the final manuscript.

## Conflict of Interest Statement

The authors declare that the research was conducted by the support of DBT, Government of India.
