# Supplementary material for: Diverse Rice Landraces of North-East India Enables the Identification of Novel Genetic Resources for Magnaporthe Resistance
Source: Front Plant Sci. 2017 Aug 29;8:1500. doi: 10.3389/fpls.2017.01500 (PMC5583601; doi:10.3389/fpls.2017.01500)
Supplement: Supplementary file 1 [file Table_1.DOCX]

**Supplementary Table S1:** Representation of gene profiling in landraces and their phenotyping to blast disease (R-resistant and S-susceptible); Grouping of landraces into corresponding subgroups by structure analysis.

| **S. NO.** | **IRGC number** | **Landrace** | **Source**  **(District/State)** | **Blast resistance** | | | **Blast Genes** | **Structural Classification** |
| --- | --- | --- | --- | --- | --- | --- | --- | --- |
|  |  |  |  | **Leaf** | **Neck** | **Leaf and Neck** |  |  |
| 1 | - | Azhoghi | Zunheboto/Nagaland | R | S | S | *Piz, Pizt* | SG2 |
| 2 | - | Nungshangphou | Bishnupur/Manipur | S | S | S | *Piz, Pizt* | SG2 |
| 3 | - | Chingtui | Churachandpur/Manipur | R | S | S | *Pi54, Pizt* | SG2 |
| 4 | - | Area old Seed | Imphal West /Manipur | R | R | R | *Pi9, Piz* | SG2 |
| 5 | - | Rosolia | Kohima/Nagaland | S | S | S | *-* | SG2 |
| 6 | - | Badshahbhog Joha | Jorhat/Assam | S | R | S | *Pi9, Pi54* | SG1 |
| 7 | - | Shamphai | Chandel/Manipur | R | R | R | *Pi9, Pi38* | SG2 |
| 8 | - | Ebe mestsi | Wokha/Nagaland | R | S | S | *Pi9, Pi54, Pita2* | SG1 |
| 9 | - | Manom tok | Wokha/Nagaland | S | S | S | *-* | AD |
| 10 | - | Kerebe phek | Phek/Nagaland | R | R | R | *Pi1, Pi9, Pi38, Pi54* | SG1 |
| 11 | - | Naga special | Wokha/Nagaland | S | R | S | *Piz* | SG2 |
| 12 | - | Churhchandpur | Churhchandpur/Manipur | R | S | S | *Pi9* | SG2 |
| 13 | 265544 | Meghalaya lakang | Shilong/Meghalaya | R | R | R | *-* | SG2 |
| 14 | - | Chakhao lamhing | Chandel/Manipur | R | R | R | *Pi9, Pi38, Pi54, Pib* | AD |
| 15 | - | Kunta mah | Ukhrul/Manipur | R | R | R | *Pi9, Piz, Pizt* | SG2 |
| 16 | 352870 | Chingphou | Ukhrul/Manipur | R | S | S | *Pi20, Piz, Pizt* | SG2 |
| 17 | - | Bizor II | Ukhurl/Manipur | S | R | S | *Piz, Pizt* | SG2 |
| 18 | - | Ato kunupu | Zunheboto/Nagaland | R | R | R | *Pi9, Pi38, Pi54, Piz* | AD |
| 19 | - | Kueashu | Zunheboto/Nagaland | S | R | S | *Piz* | SG2 |
| 20 | - | Sumi special | Wokha/Nagaland | S | S | S | *Piz, Pizt* | SG2 |
| 21 | 306333 | Chakhao amubi | Bishnupur/Manipur | S | R | S | *-* | SG2 |
| 22 | - | Ssl wonder rice | Wokha/Nagaland | R | S | S | *Pi9, Piz* | AD |
| 23 | - | Medhong tssok | Wokha/Nagaland | R | R | R | *Pi9, Pi33, Pita2* | SG2 |
| 24 | 324091 | Boha Thulasi Joha | Jorhat/Assam | R | S | S | *Pi9, Piz, Pizt* | SG2 |
| 25 | - | Kaoyeng | Churachandpur/Manipur | R | S | S | *Pi9* | SG2 |
| 26 | 521506 | Daramphou | Imphal West/Manipur | R | S | S | *Pi1, Pi54, Pita2* | SG2 |
| 27 | - | Bokachakua | Ukhrul/Manipur | S | S | S | *-* | SG2 |
| 28 | 423118 | Sars | Kohima/Nagaland | R | S | S | *-* | SG2 |
| 29 | - | Meitidak | Peren/Nagaland | R | R | R | *Piz, Pizt* | SG2 |
| 30 | 306355 | Moirangphou angovba | Bishnupur/Manipur | S | S | S | *-* | SG2 |
| 31 | - | Makhara-2 | Ukhrul/Manipur | R | R | R | *Pi9, Piz* | SG2 |
| 32 | - | Moirangphou khongnembi | Bishnupur/Manipur | R | R | R | *Pi33, Pita2, Pizt* | SG2 |
| 33 | - | Phungcham mah | Churachandpur/Manipur | R | R | R | *Pi9, Piz* | SG2 |
| 34 | - | Kapang | Ukhrul/Manipur | S | R | S | *Pi38* | SG2 |
| 35 | - | Keneui | Phek/Nagaland | R | R | R | *Pi33, Pi38, Pizt* | SG2 |
| 36 | 557482 | Niphuthokpi | Ukhrul/Manipur | R | R | R | *Pi9, Pi33, Pizt* | SG2 |
| 37 | - | Leishing phou | Imphal East/Manipur | R | S | S | *Pi9, Pi20, Pizt* | SG2 |
| 38 | - | Kunda | Bishnupur/Manipur | S | S | S | *Pi9* | SG2 |
| 39 | 464296 | Remi | West Siang/Arunachal Pradesh | R | S | S | *Pi9, Pi38, Pita2, Pizt* | SG2 |
| 40 | - | Utlou | Bishnupur/Manipur | S | S | S | *-* | SG2 |
| 41 | - | Meghalaya-lafara | Shillong/Meghalaya | S | S | S | *-* | SG2 |
| 42 | - | Arunachal | West Siang/Arunahalpradesh | R | R | R | *Pi9, Piz* | SG2 |
| 43 | - | Paikho mah | Ukhrul/Manipur | R | R | R | *Pi9, Piz* | SG2 |
| 44 | - | Makhapui kalry | Ukrhurl/Manipur | R | R | R | *Pib, Piz* | SG2 |
| 45 | - | Manui nira | Ukhrul/Manipur | R | R | R | *Pi54, Piz, Pizt* | AD |
| 46 | - | Seisonya tsia | Kohima/Nagaland | R | R | R | *Pi9, Pizt* | SG2 |
| 47 | - | Khangamra | Ukhrul/Manipur | R | R | R | *Pi9* | SG2 |
| 48 | 558244 | Bokulbora | Jorhat/Assam | S | S | S | *Pi33, Pizt* | SG2 |
| 49 | - | Allechisho | Zunheboto/Nagaland | R | R | R | *Pi9, Piz* | AD |
| 50 | - | Kbastem | Shillong/Meghalaya | S | R | S | *Piz, Pizt* | SG2 |
| 51 | - | Kapana ranta | Ukhrul/Manipur | R | S | S | *Piz* | SG2 |
| 52 | - | Haorei machang | Ukhrul/Manipur | R | S | S | *Piz* | SG2 |
| 53 | - | Manong kangbu | Kohima/Nagaland | R | S | S | *Pi9, Pib, Piz* | SG1 |
| 54 | - | Khemaru | Kohima/Nagaland | S | R | S | *Pi9, Piz* | SG1 |
| 55 | - | Glanchra | Kohima/Nagaland | R | R | R | *Pi1, Pi9, Pi54, Pita2, Piz* | SG1 |
| 56 | - | Wazhuho phek | Phek/Nagaland | R | S | S | *Pi20, Pi38, Piz* | SG2 |
| 57 | - | Rezose phek | Phek/Nagaland | R | R | R | *Pi1, Pi9* | SG2 |
| 58 | - | Kezijhum | Wokha/Nagaland | S | S | S | *-* | SG2 |
| 59 | - | Mancho tsok | Wokha/Nagaland | R | S | S | *Pi9, Pib, Piz* | SG1 |
| 60 | - | Atra | Ukhrul/Manipur | R | R | R | *Piz* | SG2 |
| 61 | - | Tamphaphou | Imphal West/Manipur | S | S | S | *Pizt* | SG2 |
| 62 | - | Bheemap eepur | Churachandpur/Manipur | S | S | S | *Pi1, Piz* | AD |
| 63 | 381847 | Mecheki | Wokha/Nagaland | R | R | R | *Pi9* | AD |
| 64 | 350461 | Chingphourel | Ukhrul/Manipur | R | R | R | *-* | SG2 |
| 65 | - | Ahutnei | Kohima/Nagaland | R | R | R | *Pi9, Pi38, Pi54, Pita2* | SG2 |
| 66 | - | Dzukenun | Kohima/Nagaland | R | S | S | *Pita2, Piz* | SG2 |
| 67 | - | Jakherpati | Ukhrul/Manipur | R | S | S | *Pi9, Pi40, Pita2, Piz, Pizt* | SG2 |
| 68 | - | Churachandpub | Churachandpur/Manipur | R | S | S | *Pi1, Pi9, Pi33, Pi54, Piz* | AD |
| 69 | - | Mipin(ad) | West Siang/Arunachal Pradesh | R | R | R | *Pi9, Pi40, Pita2* | SG2 |
| 70 | - | Menetsuk | Wokha/Nagaland | R | S | S | *Pi9* | SG2 |
| 71 | - | Kapangra | Ukhrul/Manipur | R | S | S | *Pi9, Pi38, Piz* | AD |
| 72 | - | Chinaching | Thoubal/Manipur | R | S | S | *Pi1* | SG2 |
| 73 | - | Kenijang-1 | Kohima/Nagaland | R | S | S | *Pi9, Pi54, Piz* | SG2 |
| 74 | - | Ginphou | Thoubal/Manipur | S | R | S | *Pi9, Pita2* | SG2 |
| 75 | - | Weshelora | Phek/Nagaland | S | S | S | *Pizt* | SG2 |
| 76 | 17126 | Phouren amubi | Thoubal/Manipur | S | R | S | *Piz, Pizt* | SG2 |
| 77 | - | Keda-1ss | Wokha/Nagaland | R | R | R | *Pi9, Piz, Pizt* | SG2 |
| 78 | - | M tsungi | Wokha/Nagaland | S | R | S | *Piz* | SG1 |
| 79 | - | Chinphou | Chandel/Manipur | R | R | R | *Pi9, Pi54* | SG1 |
| 80 | 394657 | Borbora | Jorhat/Assam | R | R | R | *Pi9, Pi54, Pizt* | AD |
| 81 | - | Zunhebota | Zunheboto/Nagaland | R | R | R | *Pita2, Piz, Pizt* | SG2 |
| 82 | 297608 | Erimaphou | Thoubal/Manipur | R | S | S | *Piz, Pizt* | SG2 |
| 83 | - | Tshngmekijang | Makokchung/Nagaland | S | R | S | *Pib, Piz* | SG1 |
| 84 | - | Chakhao mei | Thoubal/Manipur | S | R | S | *Piz* | SG2 |
| 85 | - | Meron tssok | Wokha/Nagaland | R | S | S | *-* | SG2 |
| 86 | - | Munichs tsok | Wokha/Nagaland | R | R | R | *Pi1, Pi9, Pi20, Piz* | AD |
| 87 | - | Desek youso | Tuensang/Nagaland | R | R | R | *Piz, Pita2* | SG2 |
| 88 | - | Talong | Ukhrul/Manipur | S | R | S | *Pi1, Pita2* | SG1 |
| 89 | - | Chakhao kumb | Bishnupur/Manipur | R | R | R | *Pi9, Pi54, Pita2, Piz* | SG1 |
| 90 | - | Zunhiboto | Zunheboto/Nagaland | R | R | R | *Pi54, Piz, Pizt* | SG2 |
| 91 | 423064 | Kolajoha | Jorhat/Assam | S | S | S | *Piz* | SG2 |
| 92 | - | Keme yaisha | Kohima/Nagaland | R | S | S | *Pi9, Pi38* | SG2 |
| 93 | - | Shangshak local | Ukhrul/Manipur | S | R | S | *-* | SG2 |
| 94 | 467112 | Phougak | Bishnupur/Manipur | R | R | R | *Pi9, Pi54, Pita2* | SG2 |
| 95 | - | Koni | Kohima/Nagland | R | S | S | *Pi9, Pi54* | SG2 |
| 96 | - | Neikedu ulhn tsia | Kohima/Nagaland | S | R | S | *Pi33* | SG2 |
| 97 | - | Naga special-2 | Kohima/Nagaland | S | R | S | *-* | SG2 |
| 98 | - | Pnal jhong | Bishnupur/Manipur | R | R | R | *Pi38, Pi54* | SG2 |
| 99 | - | Napthmei | Ukhrul/Manipur | R | S | S | *Pi9, Pi54, Piz* | SG2 |
| 100 | 394539 | Rongabora | Kohima/Nagaland | R | R | R | *Pi1, Pi9, Piz* | SG1 |
| 101 | - | Angatra | Kohima/Nagaland | S | R | S | *-* | SG2 |
| 102 | - | Konibora | Kohima/Nagaland | S | S | S | *-* | SG2 |
| 103 | - | Tssok myku | Wokha/Nagaland | R | R | R | *Pi9, Pi54* | SG2 |
| 104 | - | Tekum tssok | Wokha/Nagaland | R | R | R | *Pi9, Pitp, Piz* | SG1 |
| 105 | - | Manipur sow | Ukhrul/Manipur | R | R | R | *Pitp* | SG2 |
| 106 | - | Sapet maso | Makokchung/Nagaland | R | R | R | *Pi9* | SG2 |
| 107 | - | Moibro tsok | Wokha/Nagaland | S | R | S | *Pi9, Pi54, Pib* | SG1 |
| 108 | - | Chalhtssia | Kohima/Nagaland | R | R | R | *Pi1, Pi9, Pi54, Pib, Pitp, Piz* | SG1 |
| 109 | - | Duikungmei | Peren/Nagaland | R | R | R | *Pizt* | SG2 |
| 110 | - | Krengosa | Peren/Nagaland | S | S | S | *-* | AD |
| 111 | 297609 | Akutphou | Bishnupur/Manipur | R | R | R | *Pita2* | SG2 |
| 112 | - | Chingphourel amubi | Bishnupur/Manipur | R | S | S | *Pi9* | SG2 |
| 113 | 464571 | Chakhao poreiton | Bishnupur/Manipur | S | S | S | *Pi9, Pi54* | SG2 |
| 114 | - | Senebumap | Churachandpur/Manipur | S | S | S | *Pi38, Piz, Pizt* | AD |
| 115 | - | Matruri | Churachandpur/Manipur | S | R | S | *Pita2, Pizt* | SG2 |
| 116 | - | Kunkunijoha | Jorhat/Assam | S | S | S | *Pita2* | SG2 |
| 117 | - | Malbulow | Zunheboto/Nagaland | R | R | R | *Pi9, Pi54* | SG1 |
| 118 | - | Jungu phek | Phek/Nagaland | R | R | R | *Pi9, Pi54* | SG1 |
| 119 | - | Hasosil mah | Ukhurl/Manipur | R | R | R | *Pi9* | SG2 |
| 120 | - | Noin | Tamenglong/Manipur | S | S | S | *-* | SG2 |
| 121 | - | Sohkyrleh | Senapati/Manipur | R | S | S | *Pi9* | SG2 |
| 122 | - | Ching chakhao | Churachandpur/Manipur | S | S | S | *Pizt* | SG2 |
| 123 | - | Runya | Kohima/Nagaland | R | S | S | *Pi9, Pitp, Piz* | SG1 |
| 124 | 350774 | Laispah | Ukhurl/Manipur | R | R | R | *Pi9* | SG2 |
| 125 | - | Ayamaomaha | Senapati/Manipur | S | R | S | *Pitp, Pizt* | SG2 |
| 126 | - | Chandrel axhibition | Chandel/Manipur | S | S | S | *Pi20* | AD |
| 127 | 596561 | Longphou angouba | Thoubal/Manipur | R | S | S | *-* | SG2 |
| 128 | - | Zutsok mosta | Wokha/Nagaland | R | R | R | *Pi9, Pi38, Pitp* | AD |
| 129 | - | Ngamei | Peren/Nagaland | R | R | R | *Pi9* | SG2 |
| 130 | - | Malzu Biron | Peren/Nagaland | R | S | S | *Pi9* | SG2 |
| 131 | - | Nagaphou | Ukhrul/Manipur | R | S | S | *Pi9, Pizt* | SG2 |
| 132 | - | Hungdung | Ukhrul/Manipur | R | S | S | *Pi1, Pi9, Pi54, Pitp, Pita2* | AD |
| 133 | - | Sangbhuman | Churachandpur/Manipur | R | R | R | *Pitp, Piz* | AD |
| 134 | - | Kenhoni | Kohima/Nagaland | R | R | R | *Pizt* | SG2 |
| 135 | - | Younyo kangru | Tuensang/Nagaland | R | R | R | *Pi9, Pi54, Pitp, Piz* | AD |
| 136 | - | Bhumap | Churachandpur/Manipur | R | S | S | *Pi9, Pi20, Pi54, Piz* | SG1 |
| 137 | 465061 | Laza | Wokha/Nagaland | R | R | R | *Pi9, Pi38, Pi54, Piz* | SG1 |
| 138 | - | Yungra makori | Ukhrul/Manipur | R | R | R | *Pi1, Pi9, Pita2* | AD |
| 139 | - | Naga wonder rice | Kohima/Nagaland | R | R | R | *Pi9* | SG2 |
| 140 | - | Pumpha mah | Ukhrul/Manipur | S | S | S | *Pizt* | SG2 |
| 141 | 350525 | Machang | Ukhrul/Manipur | S | R | S | *Pita2* | SG2 |
| 142 | 423165 | Ngodzu | Kohima/Nagaland | S | R | S | *-* | SG2 |
| 143 | 410331 | Chakhao | Bishnupur/Manipur | R | S | S | *Pi9, Pita2* | SG2 |
| 144 | - | Nongrangphou | Bishnupur/Manipur | S | S | S | *Pizt* | SG2 |
| 145 | - | Kbalaispah | Shillong/Meghalaya | S | R | S | *Pi9, Pi20* | SG2 |
| 146 | - | Ngonolashia | Ukhrul/Manipur | R | R | R | *Pi9, Pi20* | SG2 |
| 147 | - | Manuikhamei | Ukhrul/Manipur | R | R | R | *-* | AD |
| 148 | - | Kemenya kepeyu | Kohima/Nagaland | R | R | R | *-* | SG2 |
| 149 | - | Otsok khira | Wokha/Nagaland | R | S | S | *Pi9, Piz* | AD |
| 150 | - | Motsuku | Zunheboto/Nagaland | S | R | S | *Piz* | SG1 |
| 151 | - | Kahangam | Senapati/Manipur | R | R | R | *Pi9, pi33* | SG2 |
| 152 | - | Nagarm mahripid | Zunheboto/Nagaland | R | S | S | *Pi9, pi33* | SG2 |
| 153 | - | Haosil mah | Ukhrul/Manipur | R | R | R | *Pi9, pi33* | SG2 |
| 154 | - | Chingtui mah | Churachandpur/Manipur | R | S | S | *Pi40, Pizt* | SG2 |
| 155 | - | Kba leihkhoo | Shillong/Meghalaya | R | R | R | *Pi33, Pi40* | SG2 |
| 156 | 465318 | Chakhao local | Bishnupur/Manipur | S | R | S | *Pi40* | SG2 |
| 157 | - | Jenil kajiephek | Phek/Nagaland | R | S | S | *Pi54, Pizt* | AD |
| 158 | - | Uteibi mah | Ukhrul/Manipur | S | R | S | *Pi40, Pizt* | SG2 |
| 159 | - | Maritchatpi | Ukhrul/Manipur | R | S | S | *Pi9, Pi40* | SG2 |
| 160 | - | Rulotsia | Kohima/Nagaland | S | R | S | *Pi40, Piz, Pizt* | SG2 |
| 161 | 464556 | Langphou angam | Churachandpur/Manipur | R | S | S | *Pi9, Pi40* | SG2 |
| 162 | - | Tangsek kangbu | Tuensang/Nagaland | S | R | S | *Pi40* | SG2 |
| 163 | - | Teke | Tuensang/Nagaland | R | R | R | *Pi9, Pi40* | AD |
| 164 | - | Podumoni Ahu | Tuensang/Nagaland | R | R | R | *Pi9, Pi40, Pita2* | AD |
| 165 | - | Epya tssok | Wokha/Nagaland | R | R | R | *Pi20, Pi40, Pi54* | SG2 |
| 166 | - | Yungra makroi | Ukhrul/Manipur | R | R | R | *Pi40, Pizt* | SG2 |
| 167 | - | Yunyokan steo | Wokha/Nagaland | R | S | S | *Pi9, Pi40, Pizt* | AD |
| 168 | 467305 | Phoutum mah | Ukhrul/Manipur | R | R | R | *Pib* | SG2 |
| 169 | 467205 | Chingpmei | Ukhrul/Manipur | R | R | R | *Pi9* | SG2 |
| 170 | 423263 | Asupa | Kohima/Nagaland | R | R | R | *Pi40 , Pib* | SG2 |
| 171 | - | Punsimutt | Kohima/Nagaland | S | S | S | *Pi40* | SG2 |
| 172 | - | Shitharia maha | Senapati/Manipur | R | R | R | *Pi40* | SG2 |
| 173 | 423303 | Apaghi june | Kohima/Nagaland | R | R | R | *Pi33, Piz, Pi9, Pib, Pi38, Pi40, Pi54* | SG1 |
| 174 | - | Kuki moso | Wokha/Nagaland | R | R | R | *Pi40, Piz* | AD |
| 175 | - | Meitak | Peren/Nagaland | S | R | S | *Pi9, pi33* | SG2 |
| 176 | - | Kenyo | Peren/Nagaland | R | S | S | *Pi9, Pi54, Pib* | SG1 |
| 177 | - | Chinapati | Senapati/Manipur | S | S | S | *Pi1, Pi54* | SG2 |
| 178 | - | Wainem | Senapati/Manipur | R | R | R | *-* | SG2 |
| 179 | - | Thekrulha | Kohima/Nagaland | R | R | R | *-* | SG1 |
| 180 | - | Vishku | Kohima/Nagaland | R | R | R | *Pizt* | SG2 |
| 181 | - | Retu masojang | Wokha/Nagaland | S | S | S | *Pizt* | SG2 |
| 182 | - | Apya tssok | Wokha/Nagland | S | R | S | *Pi20, Piz* | AD |
| 183 | - | Phorel utlou | Bishnupur/Manipur | S | S | S | *-* | SG2 |
| 184 | - | Tssish puri | Kohima/Nagaland | R | R | R | *Pi9, Pib, Piz* | SG1 |
| 185 | 464025 | Mesa tsuk | Zunheboto/Naglaland | R | R | R | *Pizt* | SG2 |
| 186 | - | Leisemjang | Makokchung/Nagaland | S | R | S | *-* | AD |
| 187 | 610299 | Balired | West Siang/Arunachal pradesh | S | R | S | *-* | AD |
| 188 | - | Etsaro | Phek/Nagaland | S | R | S | *Piz, Pizt* | SG2 |
| 189 | - | Thangmoi | Tuensang/Nagaland | S | R | S | *Pib* | SG1 |
| 190 | - | Kangyouh | Tuensang/Nagaland | R | S | S | *Pi20* | SG2 |
| 191 | - | Makhara masuta | Ukhrul/Manipur | S | R | S | *Pi33* | SG2 |
| 192 | 610298 | Bali white | West Siang/Arunachal Pradesh | R | S | S | *Pi9* | SG2 |
| 193 | - | Koyajang | Makokchung/Nagaland | R | R | R | *-* | AD |
| 194 | - | Wazuho phek | Phek/Nagaland | R | R | R | *Pi9, Pi38, Pi54, Piz* | SG1 |
| 195 | - | Rukhatang | Wokha/Nagaland | R | R | R | *Pi9, Piz* | AD |
| 196 | - | Rosole | Kohima/Nagaland | R | S | S | *Piz, Pib, Pi38, Pi54, Pi9* | SG1 |
| 197 | - | Phatsen | Tuensang/Nagaland | R | S | S | *Piz, Pib, Pi38, Pi54, Pi9* | SG1 |
| 198 | 462327 | Amana baw | Tuensang/Nagaland | S | S | S | *Pi40, Pi54* | SG2 |
| 199 | - | Meghalaya lefara | Shillong/Meghalaya | S | S | S | *Pi40, Pitp, Piz* | SG2 |
| 200 | - | Gumdhan | Kohima/Nagaland | S | R | S | *Piz* | SG2 |
| 201 | - | Malutawar | Kohima/Nagaland | S | R | S | *Piz* | SG2 |
| 202 | - | Ratkhara | Ukhrul/Manipur | R | R | R | *Pi33, Pib, Piz* | SG2 |
| 203 | - | Atukumupu | Zunheboto/Nagaland | R | R | R | *Pi33, Pib, Piz* | AD |
| 204 | - | Prakash utlou | Bishnupur/Manipur | R | S | S | *Pi9* | AD |
| 205 | - | Bhobu kangbu | Tuensang/Nagaland | R | R | R | *Pi38, Pitp* | SG2 |
| 206 | - | Moyajang | Tuensang/Nagaland | S | R | S | *Pi1* | AD |
| 207 | - | Ruchitra | Bishnupur/Manipur | R | R | R | *Pitp* | SG2 |
| 208 | - | Rebon | Zunheboto/Nagaland | R | S | S | *Pi9, Piz* | AD |
| 209 | - | Yengulo | Zunheboto/Nagaland | R | S | S | *Pi1, Pi9, Pi54, Pita2* | AD |
| 210 | 352896 | Charongphou | Chandel/Manipur | S | S | S | *Pi1, Pi54* | SG2 |
| 211 | - | Leikhamumei | Peren/Nagaland | R | S | S | *Pi9, Pi33, Piz* | SG2 |
| 212 | 464562 | Phouren | Bishnupur/Manipur | R | S | S | *Pi54, Piz* | SG2 |
| 213 | - | Tengubepher | Phek/Nagaland | R | R | R | *Pi40* | SG2 |
| 214 | - | Thenyouakha | Tuensang/Nagaland | R | S | S | *Pi9, Pi38, Pi40, Pitp, Piz* | SG1 |
| 215 | - | Sakbothong | Tuensang/Nagaland | R | S | S | *Pi38, Pi40* | SG2 |
| 216 | 465374 | Japanphou | Chandel/Manipur | S | S | S | *Piz* | SG2 |
| 217 | 276134 | Khamti | West Siang/Arunachal Pradesh | R | R | R | *Pi9, Pi33, Pitp* | SG2 |
| 218 | - | Taloimah | Ukhrul/Manipur | S | R | S | *-* | AD |
| 219 | 342359 | Saru Chakua | Chandel/Manipur | R | R | R | *Pi40* | SG2 |
| 220 | - | Bali old | West Siang/Arunachal Pradesh | S | R | S | *-* | AD |
| 221 | - | Chakhao hill | Chandel/Manipur | R | S | S | *Pi9, Pi38, Pib, Piz* | SG1 |
| 222 | - | Sercher | Imphal West/Manipur | R | S | S | *Pi9, Pi40, Piz* | SG2 |
| 223 | - | Louriphou | Imphal West/Manipur | R | S | S | *Pi40, Pitp, Pizt* | SG2 |
| 224 | - | Chingphou angouba | Ukhrul/Manipur | R | S | S | *Pi40, Piz* | AD |
| 225 | - | Khongmei mah | Ukhrul/Manipur | S | S | S | *-* | SG2 |
| 226 | - | Sanayanbi | Ukhrul/Manipur | S | S | S | *-* | SG2 |
| 227 | - | Phouren kaichang | Chandel/Manipur | S | R | S | *-* | SG2 |
| 228 | - | Kemeste | Kohima/Nagaland | R | R | R | *Pi9, Pi54, Pib, Piz* | SG1 |
| 229 | 297607 | Sanaphou | Thoubal/Manipur | R | S | S | *Pizt* | SG2 |
| 230 | 352907 | Thoibiphou | Bishnupur/Manipur | R | R | R | *Pizt* | SG2 |
| 231 | - | Yanjoepya | Wokha/Nagaland | S | R | S | *-* | AD |
| 232 | - | Deserkangbu | Tuensang/Nagaland | S | R | S | *-* | SG2 |
