# Supplementary material for: Diverse Rice Landraces of North-East India Enables the Identification of Novel Genetic Resources for Magnaporthe Resistance
Source: Front Plant Sci. 2017 Aug 29;8:1500. doi: 10.3389/fpls.2017.01500 (PMC5583601; doi:10.3389/fpls.2017.01500)
Supplement: Supplementary file 2 [file Table_2.docx]

**Supplementary Table S2:** Markers used for gene profiling of blast resistance genes.

| **Gene** | **Gene Position (Mb)** | **Positive control** | **Markers** | **Expected product size** | **Sequence** | | **Reference** |
| --- | --- | --- | --- | --- | --- | --- | --- |
|  |  |  |  |  | **Forward** | **Reverse** |  |
| *Pitp* | 25.1-28.6 | Tetep | RM246 | 116 | gagctccatcagccattcag | ctgagtgctgctgcgact | Barman *et al*., 2004 |
|  |  |  | RM3475 | 150 | gtcggtttgcctagttgagc | ttcctcggtgtatgggtctc | Barman *et al*., 2004 |
| *Pi33* | 5.9-6.1 | IR64 | RM72 | 166 | ccggcgataaaacaatgag | gcatcggtcctaactaaggg | Berruyer *et al*., 2003 |
| *Pi54* | 24.7 | Tetep | RM206 | 147 | cccatgcgtttaactattct | cgttccatcgatccgtatgg | Sharma *et al*. 2002 |
|  |  |  | RM224 | 157 | ctcgatcgatcttcacgagg | tgctataaaaggcattcggg | Fjellstrom *et al*. 2004 |
|  |  |  | PikhMAS* | 216 | caatctccaaagttttcagg | gcttcaatcactgctagacc | Ramkumar *et al*., 2011 |
| *Pib* | 35.1 | IRBL b-B/EC565171 | RM166 | 321 | ggtcctgggtcaataattgggttacc | ttgctgcatgatcctaaaccgg | Fjellstrom *et al*. 2004 |
|  |  |  | RM208 | 173 | tctgcaagccttgtctgatg | taagtcgatcattgtgtggacc | Fjellstrom *et al*. 2004 |
| *Pi20* | 6.9-10.6 | IR24 | RM1337 | 210 | gctgaggagtatcctttctc | accataggaagatcatcaca | Li *et al*. 2008 |
|  |  |  | RM5364 | 148 | gtattacgctcgatagcggc | gtatcctttctcgcaatcgc | Li *et al*. 2008 |
| *Pi38* | 19.1-21.9 | Tadukan | RM21 | 157 | acagtattccgtaggcacgg | gctccatgagggtggtagag | Gowda *et al*. 2006 |
|  |  |  | RM3605 | 107 | gatggacgacgagtagtggg | ctctccatttttccccttcc | Gowda *et al*. 2006 |
| *Pita2* | 10.0-13.2 | IRBLta2-p1[LT] | RM7102 | 169 | ttgagagcgtttttaggatg | tcggtttacttggttactcg | Fjellstrom *et al*. 2004 |
|  |  |  | RM27970 | 151 | tccaccactctgacgtctactaacc | ctgcgggaagtgtaggagaagc | Hayashi *et al*., 2006 |
|  |  |  | YL155/87^ | 1,042 | agcaggttataagctaggcc | ctaccaacaagttcatcaaa | Jia *et al*., 2002, 2004 |
| *Pi1* | 26.4-28.3 | C101LAC | RM224 | 157 | atcgatcgatcttcacgagg | tgctataaaaggcattcggg | Hittalmani *et al*., 2000 |
| *Piz* | 10.1-10.5 | Zenith | RM19818 | 275 | aaccctagactactcccggtctcc | accttggcgagctctgtttcg | Zhou *et al*. 2006 |
|  |  |  | AP56595¥ | 288 | ctccttcagctgctcctc | tgatgacttccaaacggtag | Fjellstrom *et al*., 2006 |
|  |  |  | Z56592# | 292 | ggacccgcgttttccacgtgtaa | aggaatctattgctaagcatgac | Hayashi *et al*., 2006 |
| *Pi9* | 10.3 | IRBL9-W[LT] | RM7213 | 211 | ctatagccagcgacgaggac | ctgcacccatctctctctcc | Qu *et al*. 2006 |
|  |  |  | 195R-1^ | 2000 | atggtcctttatctttattg | ttgctccatctcctctgtt | Qu *et al*., 2006 |
|  |  |  | Nmsmpi9-1¥ | 168 | cgagaaggacatctggtacg | gagatgcttggatttagaagac | Devi *et al*., 2015 |
| *Pizt* | 10.1-10.5 | IRBLb-B[LT] | Zt56591# | 257 | ttgctgagccattgttaaaca | atctcttcatatatatgaaggccac | Hayashi *et al*., 2006 |
| *Pi40* | 16.2-17.5 | IR65482-4-1-136-2-2 | MSM6¥ | 256 | tgctgagatagccgagaaatc | gcacccttttcgctagagg | Devi *et al*., 2015 |

Markers - *Indel, #SNP, ^gene specific STS and ¥ allele specific
