# Supplementary material for: Diverse Rice Landraces of North-East India Enables the Identification of Novel Genetic Resources for Magnaporthe Resistance
Source: Front Plant Sci. 2017 Aug 29;8:1500. doi: 10.3389/fpls.2017.01500 (PMC5583601; doi:10.3389/fpls.2017.01500)
Supplement: Supplementary file 3 [file Table_3.docx]

**Supplementary Table S3**: Diversity analysis of landraces.

| **Marker** | **Chromosome** | **Physical location (Mb)** | **Allele No** | **Major Allele Frequency** | **Observed Heterozygosity (*Ho*)** | **Gene Diversity**  **(*He*)** | **PIC** |
| --- | --- | --- | --- | --- | --- | --- | --- |
| RM10038 | 1 | 0.6 | 5 | 0.53 | 0.08 | 0.62 | 0.56 |
| RM151 | 1 | 5.5 | 3 | 0.42 | 0.09 | 0.53 | 0.55 |
| RM3627 | 1 | 10.3 | 4 | 0.39 | 0.06 | 0.72 | 0.67 |
| RM7075 | 1 | 15.1 | 4 | 0.42 | 0.05 | 0.65 | 0.58 |
| RM11111 | 1 | 20.3 | 3 | 0.53 | 0.09 | 0.52 | 0.32 |
| RM11307 | 1 | 24.2 | 2 | 0.41 | 0.09 | 0.63 | 0.28 |
| RM3336 | 1 | 28.6 | 4 | 0.33 | 0.08 | 0.74 | 0.69 |
| RM302 | 1 | 32.9 | 4 | 0.35 | 0.08 | 0.71 | 0.66 |
| RM11865 | 1 | 36.2 | 3 | 0.32 | 0.09 | 0.44 | 0.32 |
| RM5794 | 1 | 41.4 | 2 | 0.36 | 0.08 | 0.35 | 0.23 |
| RM485 | 2 | 0.9 | 3 | 0.72 | 0.07 | 0.43 | 0.38 |
| RM12585 | 2 | 4.5 | 3 | 0.53 | 0.05 | 0.55 | 0.29 |
| RM1358 | 2 | 10.1 | 4 | 0.51 | 0.03 | 0.64 | 0.59 |
| RM13131 | 2 | 14.6 | 3 | 0.69 | 0.12 | 0.61 | 0.32 |
| RM13263 | 2 | 18.1 | 2 | 0.61 | 0.09 | 0.68 | 0.25 |
| RM13495 | 2 | 22.2 | 2 | 0.53 | 0.03 | 0.5 | 0.37 |
| RM573 | 2 | 27.9 | 3 | 0.52 | 0.08 | 0.42 | 0.32 |
| RM13958 | 2 | 31.2 | 3 | 0.71 | 0.12 | 0.34 | 0.62 |
| RM208 | 2 | 35.1 | 5 | 0.4 | 0.14 | 0.69 | 0.53 |
| RM6137 | 2 | 35.7 | 2 | 0.5 | 0.09 | 0.5 | 0.38 |
| RM523 | 3 | 1.3 | 2 | 0.82 | 0.08 | 0.3 | 0.25 |
| RM489 | 3 | 4.3 | 3 | 0.76 | 0.16 | 0.37 | 0.31 |
| RM14765 | 3 | 9.8 | 3 | 0.72 | 0.08 | 0.47 | 0.28 |
| RM14898 | 3 | 12.43 | 3 | 0.53 | 0.09 | 0.45 | 0.43 |
| RM15111 | 3 | 15.5 | 2 | 0.59 | 0.1 | 0.48 | 0.37 |
| RM8208 | 3 | 22.2 | 3 | 0.57 | 0.07 | 0.62 | 0.46 |
| RM15630 | 3 | 26 | 2 | 0.69 | 0.04 | 0.42 | 0.33 |
| RM15766 | 3 | 28.1 | 2 | 0.68 | 0.06 | 0.53 | 0.32 |
| RM3719 | 3 | 32.42 | 4 | 0.62 | 0.02 | 0.48 | 0.37 |
| RM16162 | 3 | 35 | 2 | 0.54 | 0.09 | 0.32 | 0.33 |
| RM537 | 4 | 0.1 | 3 | 0.74 | 0.12 | 0.46 | 0.36 |
| RM6487 | 4 | 4.6 | 3 | 0.44 | 0.22 | 0.64 | 0.57 |
| RM16559 | 4 | 9.32 | 2 | 0.55 | 0.09 | 0.56 | 0.47 |
| RM16649 | 4 | 13.5 | 4 | 0.58 | 0.02 | 0.57 | 0.51 |
| RM16825 | 4 | 18.5 | 3 | 0.66 | 0.08 | 0.66 | 0.38 |
| RM1142 | 4 | 21.5 | 3 | 0.55 | 0.04 | 0.59 | 0.53 |
| RM3785 | 4 | 24.2 | 4 | 0.83 | 0.02 | 0.3 | 0.28 |
| RM17377 | 4 | 28.9 | 2 | 0.79 | 0.14 | 0.41 | 0.48 |
| RM17473 | 4 | 31 | 3 | 0.68 | 0.09 | 0.52 | 0.53 |
| RM280 | 4 | 34.9 | 4 | 0.58 | 0.1 | 0.58 | 0.52 |
| RM122 | 5 | 0.3 | 3 | 0.52 | 0.09 | 0.48 | 0.59 |
| RM437 | 5 | 3.8 | 3 | 0.45 | 0.37 | 0.64 | 0.56 |
| RM7118 | 5 | 6 | 2 | 0.64 | 0.02 | 0.46 | 0.35 |
| RM5844 | 5 | 9 | 2 | 0.66 | 0.12 | 0.67 | 0.66 |
| RM5140 | 5 | 13.4 | 3 | 0.55 | 0.09 | 0.51 | 0.28 |
| RM18516 | 5 | 17.3 | 3 | 0.48 | 0.06 | 0.61 | 0.53 |
| RM5401 | 5 | 22.1 | 2 | 0.62 | 0.02 | 0.47 | 0.36 |
| RM6334 | 5 | 24.9 | 2 | 0.56 | 0.13 | 0.71 | 0.54 |
| RM480 | 5 | 27.3 | 4 | 0.94 | 0.04 | 0.12 | 0.12 |
| RM6015 | 5 | 29.5 | 3 | 0.59 | 0.08 | 0.32 | 0.44 |
| RM190 | 6 | 1.7 | 2 | 0.55 | 0.08 | 0.5 | 0.37 |
| RM3370 | 6 | 6.6 | 3 | 0.53 | 0.01 | 0.52 | 0.41 |
| RM5963 | 6 | 8.8 | 2 | 0.43 | 0.02 | 0.43 | 0.58 |
| RM3635 | 6 | 11.1 | 4 | 0.38 | 0.08 | 0.72 | 0.67 |
| RM19976 | 6 | 13.3 | 3 | 0.39 | 0.08 | 0.44 | 0.59 |
| RM20096 | 6 | 17.2 | 2 | 0.63 | 0.12 | 0.54 | 0.62 |
| RM3827 | 6 | 21.9 | 3 | 0.68 | 0.02 | 0.45 | 0.37 |
| RM20536 | 6 | 26.7 | 3 | 0.76 | 0.08 | 0.65 | 0.65 |
| RM494 | 6 | 28.21 | 2 | 0.77 | 0.13 | 0.36 | 0.29 |
| RM103 | 6 | 30.3 | 3 | 0.68 | 0.13 | 0.56 | 0.58 |
| RM21052 | 7 | 3.7 | 2 | 0.98 | 0 | 0.05 | 0.05 |
| RM21206 | 7 | 6.3 | 4 | 0.75 | 0.09 | 0.43 | 0.68 |
| RM5499 | 7 | 10 | 5 | 0.78 | 0.07 | 0.38 | 0.35 |
| RM21435 | 7 | 13.2 | 4 | 0.58 | 0.08 | 0.23 | 0.48 |
| RM5481 | 7 | 16.1 | 3 | 0.49 | 0.12 | 0.19 | 0.59 |
| RM432 | 7 | 18.9 | 4 | 0.71 | 0.02 | 0.45 | 0.41 |
| RM5847 | 7 | 23.5 | 5 | 0.33 | 0.09 | 0.73 | 0.68 |
| RM21976 | 7 | 25.5 | 3 | 0.43 | 0.09 | 0.52 | 0.67 |
| RM21936 | 7 | 28.6 | 3 | 0.75 | 0.07 | 0.38 | 0.31 |
| RM420 | 7 | 29.3 | 2 | 0.55 | 0.09 | 0.67 | 0.32 |
| RM6356 | 8 | 1.55 | 3 | 0.58 | 0.22 | 0.56 | 0.49 |
| RM5556 | 8 | 4.4 | 3 | 0.68 | 0.09 | 0.48 | 0.43 |
| RM22674 | 8 | 8.4 | 3 | 0.76 | 0.12 | 0.48 | 0.33 |
| RM22837 | 8 | 12.3 | 8 | 0.31 | 0.04 | 0.8 | 0.77 |
| RM22971 | 8 | 16.7 | 3 | 0.66 | 0.09 | 0.54 | 0.43 |
| RM22997 | 8 | 17.6 | 4 | 0.54 | 0.08 | 0.67 | 0.54 |
| RM515 | 8 | 20.2 | 4 | 0.38 | 0.06 | 0.69 | 0.63 |
| RM23251 | 8 | 22.4 | 3 | 0.44 | 0.13 | 0.53 | 0.32 |
| RM149 | 8 | 24.7 | 3 | 0.43 | 0.12 | 0.66 | 0.65 |
| RM4997 | 8 | 28.2 | 2 | 0.9 | 0.07 | 0.18 | 0.16 |
| RM23671 | 9 | 0.6 | 3 | 0.45 | 0.11 | 0.23 | 0.54 |
| RM23800 | 9 | 4.5 | 3 | 0.44 | 0.13 | 0.28 | 0.29 |
| RM23946 | 9 | 7.8 | 4 | 0.6 | 0.17 | 0.56 | 0.5 |
| RM3912 | 9 | 10.8 | 3 | 0.76 | 0.08 | 0.43 | 0.52 |
| RM6051 | 9 | 12.7 | 3 | 0.48 | 0.06 | 0.56 | 0.46 |
| RM6839 | 9 | 14.5 | 2 | 0.72 | 0.09 | 0.54 | 0.43 |
| RM6532 | 9 | 16.6 | 5 | 0.5 | 0.94 | 0.64 | 0.58 |
| RM3787 | 9 | 19.7 | 3 | 0.44 | 0.06 | 0.65 | 0.58 |
| RM24654 | 9 | 20.1 | 3 | 0.58 | 0.03 | 0.49 | 0.37 |
| RM24842 | 9 | 22.8 | 2 | 0.55 | 0.09 | 0.71 | 0.32 |
| RM5095 | 10 | 0 | 3 | 0.79 | 0.11 | 0.69 | 0.23 |
| RM6404 | 10 | 2.4 | 2 | 0.91 | 0.02 | 0.17 | 0.15 |
| RM25104 | 10 | 5.5 | 4 | 0.43 | 0.12 | 0.23 | 0.34 |
| RM10435 | 10 | 7.1 | 3 | 0.46 | 0.04 | 0.61 | 0.53 |
| RM1126 | 10 | 9.6 | 2 | 0.6 | 0.02 | 0.48 | 0.37 |
| RM25289 | 10 | 11.1 | 3 | 0.75 | 0.08 | 0.23 | 0.29 |
| RM25421 | 10 | 14.2 | 2 | 0.66 | 0.05 | 0.33 | 0.58 |
| RM171 | 10 | 18.7 | 3 | 0.65 | 0.08 | 0.31 | 0.56 |
| RM147 | 10 | 20.9 | 2 | 0.69 | 0 | 0.42 | 0.33 |
| RM228 | 10 | 22.2 | 3 | 0.45 | 0.15 | 0.65 | 0.57 |
| RM286 | 11 | 0.3 | 3 | 0.72 | 0.1 | 0.42 | 0.35 |
| RM26213 | 11 | 4.7 | 2 | 0.77 | 0.12 | 0.64 | 0.42 |
| RM536 | 11 | 8.8 | 2 | 0.56 | 0.08 | 0.67 | 0.29 |
| RM287 | 11 | 13.7 | 3 | 0.43 | 0.02 | 0.62 | 0.54 |
| RM26656 | 11 | 15.1 | 2 | 0.54 | 0.04 | 0.71 | 0.32 |
| RM209 | 11 | 17.8 | 4 | 0.43 | 0.05 | 0.62 | 0.54 |
| RM26921 | 11 | 20.1 | 3 | 0.38 | 0.04 | 0.58 | 0.56 |
| RM6105 | 11 | 23.2 | 2 | 0.81 | 0.08 | 0.31 | 0.26 |
| RM27172 | 11 | 24.7 | 2 | 0.79 | 0.03 | 0.42 | 0.32 |
| RM2136 | 11 | 28.4 | 3 | 0.54 | 0.12 | 0.56 | 0.47 |
| RM5927 | 12 | 2.2 | 4 | 0.43 | 0.08 | 0.65 | 0.58 |
| RM5746 | 12 | 5 | 2 | 0.83 | 0.04 | 0.29 | 0.25 |
| RM27840 | 12 | 8 | 2 | 0.72 | 0.12 | 0.43 | 0.32 |
| RM27973 | 12 | 12.3 | 3 | 0.43 | 0.09 | 0.47 | 0.48 |
| RM28076 | 12 | 15.1 | 2 | 0.62 | 0.06 | 0.45 | 0.38 |
| RM28157 | 12 | 17.3 | 3 | 0.53 | 0.08 | 0.56 | 0.47 |
| RM28404 | 12 | 21.8 | 7 | 0.81 | 0.07 | 0.33 | 0.31 |
| RM2854 | 12 | 23.6 | 2 | 0.68 | 0.1 | 0.43 | 0.48 |
| RM1159 | 12 | 25.9 | 2 | 0.76 | 0.08 | 0.54 | 0.66 |
| RM1227 | 12 | 27.3 | 3 | 0.66 | 0.14 | 0.39 | 0.42 |
| **MEAN** |  |  | **3** | **0.59** | **0.09** | **0.5** | **0.44** |
