# Supplementary material for: Diverse Rice Landraces of North-East India Enables the Identification of Novel Genetic Resources for Magnaporthe Resistance
Source: Front Plant Sci. 2017 Aug 29;8:1500. doi: 10.3389/fpls.2017.01500 (PMC5583601; doi:10.3389/fpls.2017.01500)
Supplement: Supplementary file 4 [file Table_4.docx]

**Supplementary Table S4:**

**(S4A)** Coreset of North East landraces demarcated by using Morphological and molecular markers.

| **S. NO.** | **Core landraces** |
| --- | --- |
| 6 | Badshahbhog Joha |
| 9 | Manom tok |
| 13 | Meghalaya lakang |
| 47 | Khangamra |
| 49 | Allechisho |
| 53 | Manong kangbu |
| 62 | Bheemap eepur |
| 64 | Chingphourel |
| 78 | M tsungi |
| 82 | Erimaphou |
| 107 | Moibro tsok |
| 109 | Duikungmei |
| 110 | Krengosa |
| 112 | Chingphourelamubi |
| 117 | Malbulow |
| 132 | Hungdung |
| 147 | Manuikhamei |
| 148 | Kemenya kepeyu |
| 163 | Teke |
| 164 | Podumoni Ahu |
| 178 | Wainem |
| 179 | Thekrulha |
| 184 | Tssish puri |
| 191 | Makhara masuta |
| 193 | Koyajang |
| 194 | Wazuho phek |
| 197 | Phatsen |
| 201 | Malutawar |
| 202 | Ratkhara |
| 214 | Thenyouakha |
| 228 | Kemeste |
| 231 | Yanjoepya |
| 232 | Deserkangbu |

(S**4B)** Non-heuristic values of identified coreset.

| **Powercore** |  |
| --- | --- |
| Efficiency index | 0.82 |
| Mean difference% | 4.42 |
| Coincidence rate% | 100 |
| Variance difference% | 29.17 |
| Variable rate% | 118.18 |
| PIC | 0.99 |
| C-Count | 33 |
| E-Count | 232 |
