# Supplementary figures and images for: Diverse Rice Landraces of North-East India Enables the Identification of Novel Genetic Resources for Magnaporthe Resistance
Source: Front Plant Sci. 2017 Aug 29;8:1500. doi: 10.3389/fpls.2017.01500 (PMC5583601; doi:10.3389/fpls.2017.01500)

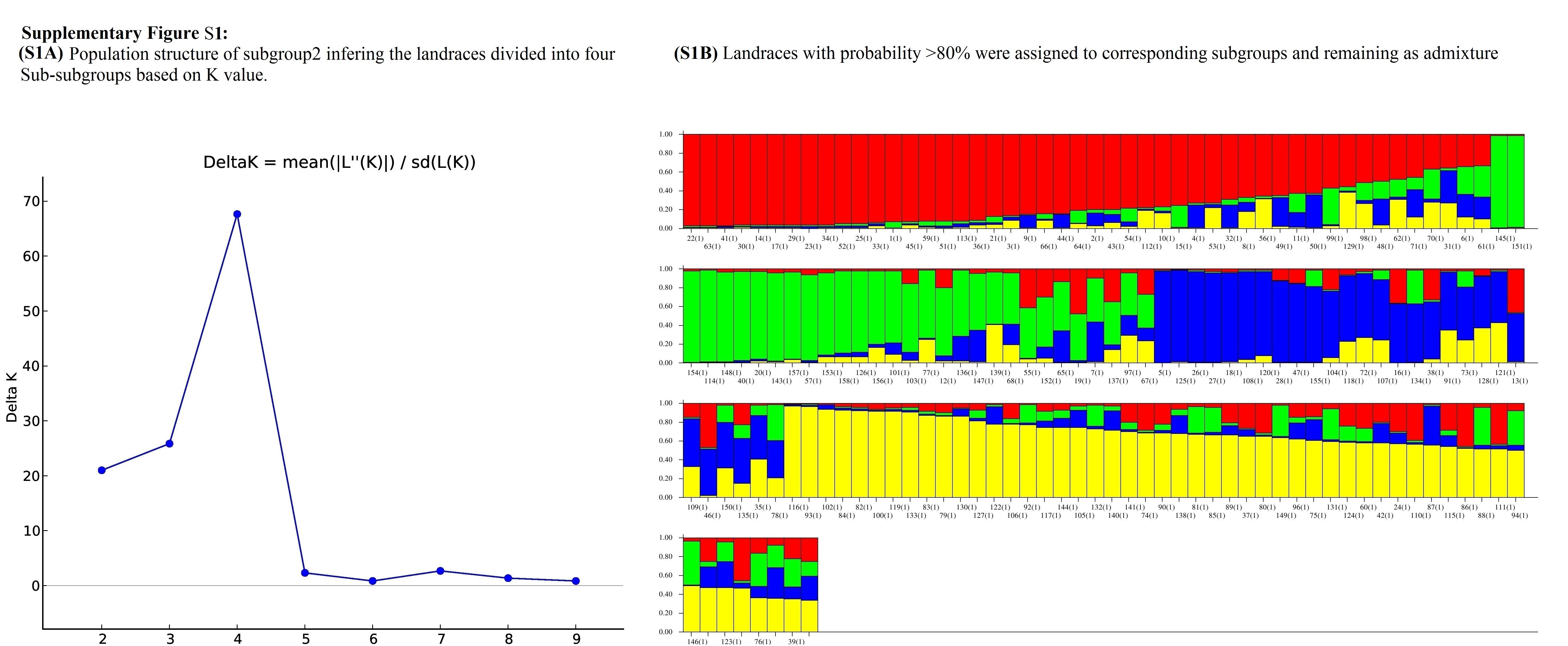

Supplement: Supplementary file 5 [file Image_1.JPEG]

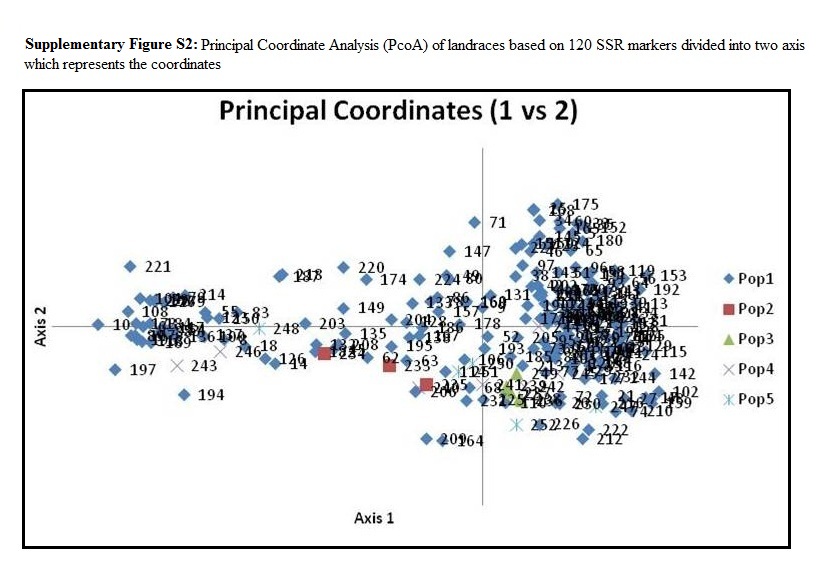

Supplement: Supplementary file 6 [file Image_2.JPEG]

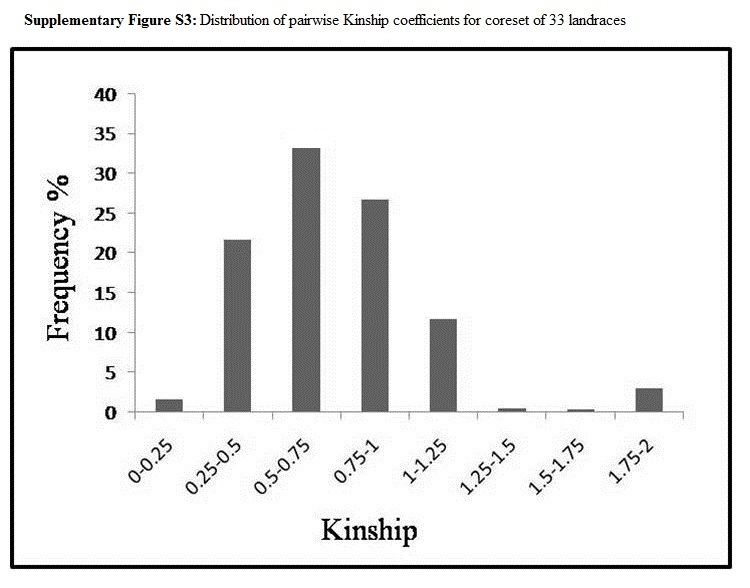

Supplement: Supplementary file 7 [file Image_3.JPEG]
